# Flexural Response of Axially Restricted RC Beams: Numerical and Theoretical Study

**DOI:** 10.3390/ma15176052

**Published:** 2022-09-01

**Authors:** Han Hu, Sergio M. R. Lopes, Adelino V. Lopes, Tiejiong Lou

**Affiliations:** 1 School of Civil Engineering and Architecture, Wuhan University of Technology, Wuhan 430070, China; 2 CEMMPRE, Department of Civil Engineering, University of Coimbra, 3030-788 Coimbra, Portugal; 3 INESC, Department of Civil Engineering, University of Coimbra, 3030-788 Coimbra, Portugal

**Keywords:** flexural behavior, axial restriction, beam, numerical analysis

## Abstract

Reinforced concrete (RC) frame beams are subject to axial restriction at the ends, which plays an important role in the nonlinear behavior of these beams. This paper presents a numerical and theoretical investigation into the flexural behavior of RC beams axially restricted with external steel or fiber reinforced polymer (FRP) reinforcement. A numerical procedure for RC beams axially restricted with external reinforcement has been developed and it is verified against available experimental results. A numerical parametric study is then performed on axially restricted RC beams, focusing on the effect of type, area, and depth of external reinforcement. The results show that axial restriction increases the post-cracking stiffness and ultimate load-carrying capacity but reduces the flexural ductility. The ultimate stress in external reinforcement is substantially impacted by reinforcement type, area, and depth. A simplified model is developed to predict the ultimate load of RC beams axially restricted with external steel/FRP reinforcement. The predictions of the proposed simplified model agree favorably with the numerical results. The correlation coefficient for the ultimate load is 0.984, and the mean difference is −2.11% with a standard deviation of 3.62%.

## 1. Introduction

Reinforced concrete (RC) beams in a frame context are axially restricted by the connections to other structural elements [1]. When the beams are elongated under transverse loading, the axial restriction would produce a compression, with a level depending on both the restriction stiffness and the beam elongation [2,3]. It has been demonstrated that when a beam experiences medium deformations, this compression can lead to axial stresses comparable to stresses induced by transverse loading [4,5]. Therefore, the axial restriction plays a critical role in the nonlinear behavior of axially restricted RC beams. However, in codes of practice [6], the effect of axial restriction is commonly neglected in the design of RC beams that are axially restricted.

A set of theoretical works [7,8,9] on axially restricted RC beams under torsion have been carried out in the University of Beira Interior. In 2015, Bernardo et al. [7] developed a theoretical model for predicting the torsional behavior of axially restricted RC beams. A modified variable angle truss model was utilized to take into consideration the impact of compressive stresses induced by axial restricting. Their model was verified with available experimental data and against numerical data of finite element analysis. In a later study [8], this model was applied to investigate the ultimate torsional performance of axially restricted RC beams with squared cross section. A parametric analysis was conducted to evaluate the influence of various important variables, i.e., the torsional reinforcement ratio, the concrete grade, and the axial restricting level. In addition, a regression analysis was performed and practical design charts for axially restricted RC beams under torsion was proposed. Further investigation was performed in 2018 on axially restricted RC rectangular beams [9]. In addition to the three parameters involved in their previous study, the height-to-width ratio was also considered in the parametric study. Based on the nonlinear regression analysis, new charts were developed for the torsional design of axially restricted RC rectangular beams.

Luo et al. [10] conducted cyclic lateral loading tests on four RC beam-column specimens to study the influence of axial restriction on beam bending and joint shear performance. To experimentally simulate the passive axial compression force in RC frame beams, they connected a self-restraint system to the beams. The experimental results showed that the axial restriction substantially improved the flexural capacity of the frame beams. At 3% drift, the flexural capacities were enhanced by 141–155% in the case of positive bending, and by 74–86% in the case of negative bending. Mihaylov et al. [11] carried out laboratory tests consisting of four large coupled beams with different axial restriction degrees and loading types. A strut-and-tie model was also proposed to analyze the test results. Their study indicated that axial restriction generated high compression, hence altering substantially the cracking mode and shear performance of the beams. Poudel et al. [12] studied experimentally the effect of passive axial restrictions on the performance of RC coupling beams. It was shown that the axially restricted beams were stronger than the unrestricted counterparts. The ACI building code greatly underestimated the strength of diagonally RC coupling beams, especially in the presence of axial restriction.

More recently, Thienpont et al. [13] performed an experimental study to examine the effect of axial restriction on the flexural behavior of hollow core RC slabs. Their tests consisted of two axially restricted specimens and one unrestricted reference specimen. The test results showed that axially restricted specimens exhibited significantly higher load-carrying capacities but smaller ultimate deflections when compared to the unrestricted reference specimen. In addition, a finite element model was developed by using ABAQUS to simulate the behavior of hollow core slabs. The numerical results showed that the tensile strength of concrete had an important influence on the load-carrying capacities of axially restricted slabs.

RC beams in flexure are expected to experience noticeable elongations during the post-cracking stage. If the beams are axially restricted, the compression at the ends introduced by axial restriction is increasingly notable with the development of member deformation. The effect of axial restriction can be simulated by using external reinforcement connected to the RC beam at the ends [14]. The external reinforcement is usually placed over the tensile zone. So, tensile forces develop in external reinforcement when the beam deflects under transverse loading. Correspondingly, axially restricting forces induced by external reinforcement are applied on the beam. In this context, axially restricted RC beams can be considered as a particular case of RC beams with external tendons (i.e., zero initial prestress). The behavior of the latter beams has been extensively addressed [15,16,17,18]. Fiber reinforced polymer (FRP) composites are recognized as a promising alternative to steel reinforcement in reinforced [19,20,21,22] and prestressed concrete applications [23,24,25,26]. This composite reinforcement may be made of carbon FRP (CFRP) [27], aramid FRP (AFRP) [28] or glass FRP (GFRP) [29].

Few studies have so far been performed on RC beams axially restricted with external reinforcement [30]. This paper is an extension of the work presented in an international conference in Coimbra [30]. The study aims to improve the performance understanding of RC beams axially restricted with external reinforcement and to propose a practical theoretical method for predicting the load-carrying capacity of these beams. A numerical procedure has been developed to simulate the flexural response of RC beams axially restricted with external reinforcement, and numerical predictions are compared with experimental results. A numerical parametric study is then carried out, focusing on the effect of the type, area, and depth of external reinforcement. In addition, a simplified model is developed to predict the ultimate stress in external reinforcement and the ultimate load of RC beams axially restricted with external steel/FRP reinforcement.

## 2. Numerical Procedure

### 2.1. Material Laws

The stress–strain relationship for concrete in compression suggested by Hognestad [31] is adopted in this study. It is composed of a parabolic ascending branch and a linearly descending branch, as indicated by

For ascending branch,

(1) $$\sigma_{c}=f_{c}\left[\frac{2\varepsilon_{c}}{\varepsilon_{0}}-{\left(\frac{\varepsilon_{c}}{\varepsilon_{0}}\right)}^{2}\right]$$

For descending branch,

(2) $$\sigma_{c}=f_{c}\left[1-0.15\left(\frac{\varepsilon_{c}-\varepsilon_{0}}{\varepsilon_{u}-\varepsilon_{0}}\right)\right]$$

where *σ_c_* and *ε_c_* are concrete stress and strain, respectively; *f_c_* is the concrete cylinder compressive strength; *ε*_0_ is the concrete strain at peak stress, taken equal to 0.002 according to Hognestad [31]; and *ε_u_* is the ultimate concrete compressive strain. The value of *ε_u_* depends on the concrete grade and confinement condition. For normal-strength concrete without confinement, the value of *ε_u_* is 0.003 according to ACI 318-19 [6].

The stress–strain curve for concrete in tension is assumed to be composed of a linearly ascending branch before cracking and a linearly descending branch after cracking up to zero stress [32]. The stress–strain relationship is expressed by

For ascending branch,

(3) $$\sigma_{c}=E_{c}\varepsilon_{c}$$

For descending branch,

(4) $$\sigma_{c}=f_{t}\left[1-\frac{\varepsilon_{c}-\varepsilon_{cr}}{\varepsilon_{tu}-\varepsilon_{cr}}\right]$$

where *E_c_* is the concrete elastic modulus; *f_t_* is the concrete tensile strength; *ε_cr_* is the concrete cracking strain; and *ε_tu_* is the concrete tensile strain corresponding to zero stress, taken as 10 times cracking strain.

Both internal steel rebars and external steel reinforcement are assumed to be elastic-perfectly plastic in both tension and compression. FRPs are linearly elastic up to rupture.

### 2.2. Numerical Algorithm

**Figure 1:**
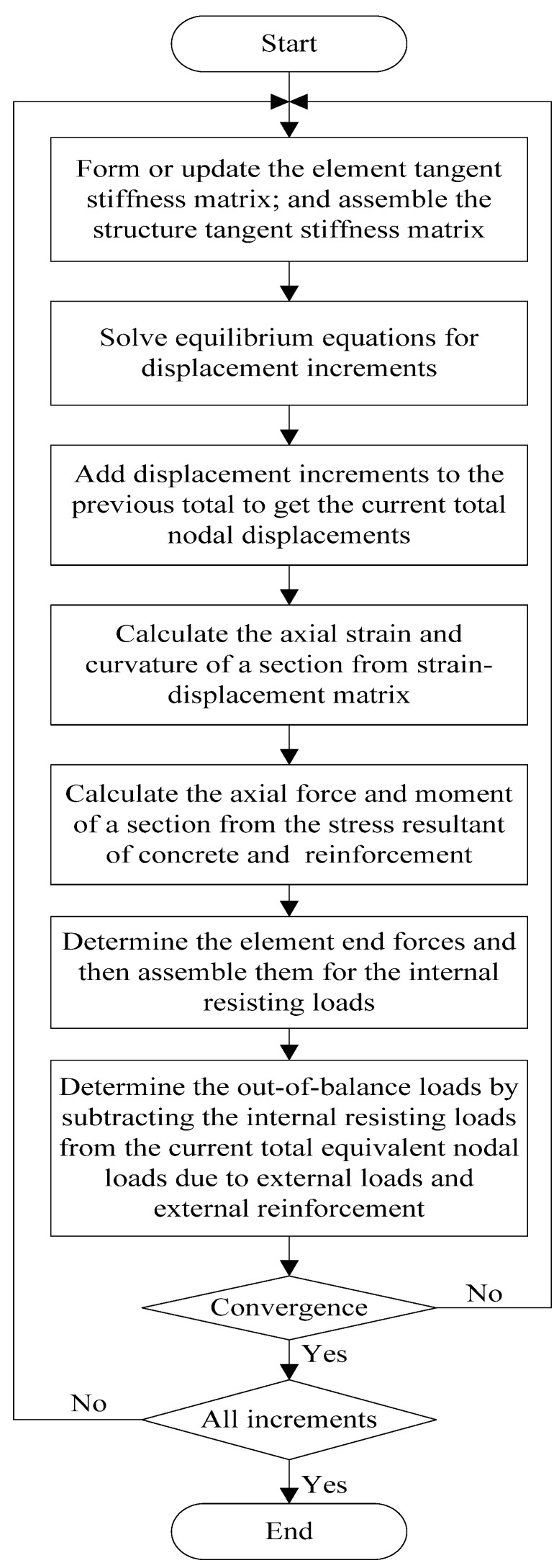
Flowchart of numerical algorithm.

The numerical model used in this study is formulated by utilizing the nonlinear beam flexural theory [33]. It is assumed that a plane section remains plane after deformations and that internal steel rebars bond perfectly with the surrounding concrete. In this model, the concrete beam is divided into a number of beam elements. The cross section of a beam element is subdivided into discrete layers to include varied material properties. The contribution of external reinforcement to the concrete beam is made by transforming the current force in external reinforcement into equivalent nodal loads applied on the beam elements [34]. The member equilibrium equations are assembled in the global coordinate system from the contributions of all the elements. After imposing appropriate boundary conditions, the nonlinear equilibrium equations for the structures are solved by the incremental-iterative method. In every increment, the Newton–Raphson iterative algorithm is used to eliminate the out-of-balance loads. The iterative procedure for each increment involves four basic steps: (1) form the current tangent stiffness matrix; (2) solve the equilibrium equations; (3) determine the current state for each element; and (4) check convergence. A flowchart illustrating the detailed solution algorithm is given in Figure 1.

The above-mentioned numerical procedure takes into account both material and geometrical nonlinearities (i.e., second-order effect as a result of the change in effective depth of external reinforcement). The proposed procedure is capable of effectively and efficiently predicting the nonlinear behavior of RC beams axially restricted with external reinforcement from zero loads up to the ultimate limit state.

### 2.3. Comparison with Experimental Data

**Figure 2:**
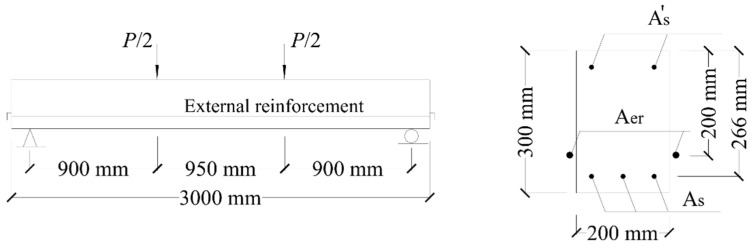
Details of test specimens.

Two beam specimens (B5 and B6) tested in Coimbra [14] are used. The beams were identical except for the axial restriction condition, i.e., B5 was a RC beam without axial restriction while B6 was a RC beam axially restricted with external steel reinforcement. The rectangular beams were simply-supported over 2750 mm in span and were subjected to two-point loading, as shown in Figure 2. The areas of bottom and top internal steel rebars, *A_s_* and $A_{s}^{\prime }$, were 339 and 101 mm^2^, respectively. The area of external steel reinforcement for Specimen B6 was 707 mm^2^. The steel yield strength was 559 MPa. The concrete cylinder compressive strength, tensile strength. And elastic modulus were 25.3 Mpa, 2.6 Mpa, and 29.1 GPa, respectively.

**Figure 3:**
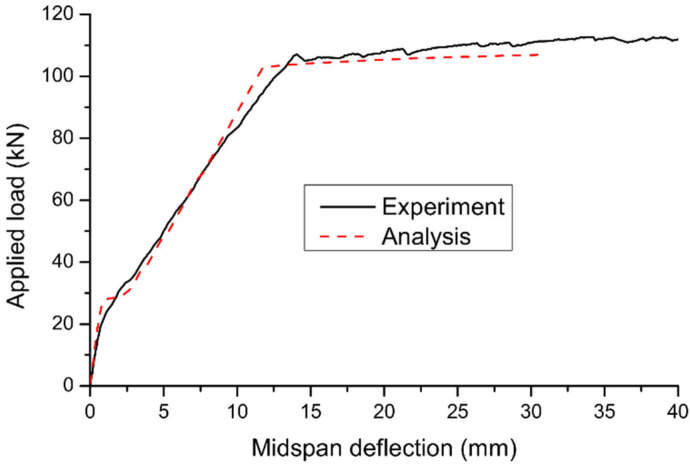
Comparison between predicted load-deflection curve and experimental data for B5.

**Figure 4:**
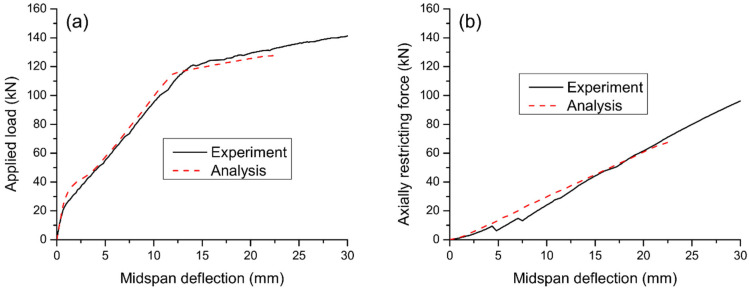
Comparison between numerical predictions and experimental data for B6. (**a**) load-deflection curve; (**b**) axially restricting force versus deflection.

According to numerical simulations, both specimens have failed due to crushing of concrete at midspan. At failure, the internal steel rebars have yielded while the external reinforcement in Specimen B6 was still in the elastic range. The above phenomena are consistent with the experimental observations. Figure 3 shows a comparison of the predicted load–deflection curve with the experimental results for Specimen B5. Good agreement between numerical and experimental results can be observed, including the cracking, yielding, and ultimate loads. However, the numerical procedure underestimates the ultimate deflection of the beam. This can be explained by the fact that the ultimate concrete compressive strain specified in the numerical procedure (i.e., *ε_u_* = 0.003) is smaller than the experimental value. For Specimen B6, the deflection versus applied load and axially restricting force relationships predicted by the numerical procedure are compared with the experimental results in Figure 4a,b, respectively. Despite some discrepancy, the numerical procedure reproduces satisfactorily the experimental results of the axially restricted RC beam.

## 3. Numerical Parametric Study

**Figure 5:**
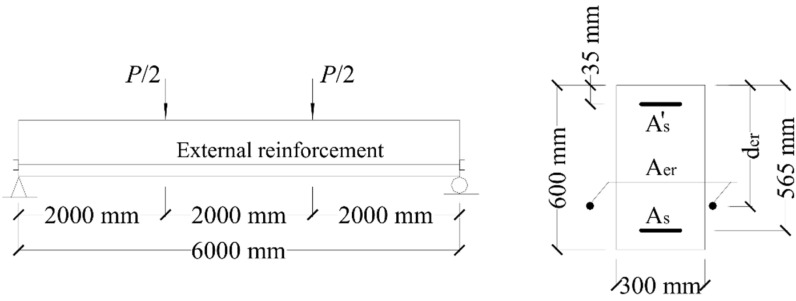
Details of the beams used for numerical evaluation.

Rectangular RC beams axially restricted with external reinforcement, as shown in Figure 5, are used to illustrate the results obtained from the analysis. The areas of bottom and top bonded steel rebars, *A_s_* and $A_{s}^{\prime }$, are 1060 and 360 mm^2^, respectively. The elastic modulus and yield strength of steel rebars are 200 GPa and 530 MPa, respectively. The cylinder compressive strength and tensile strength of concrete are 40 and 3.0 MPa, respectively. Three parameters related to external reinforcement are analyzed, i.e., the type, area (*A_er_*), and depth (*d_er_*) of external reinforcement. External reinforcement is made of steel (elastic modulus of 200 GPa and yield strength of 650 MPa), CFRP (elastic modulus of 150 GPa and rupture strength of 1840 MPa), or GFRP (elastic modulus of 40 GPa and rupture strength of 620 MPa).

### 3.1. Effect of Reinforcement Type

**Figure 6:**
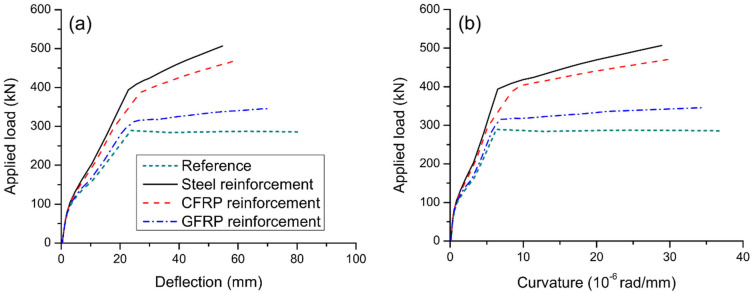
Effect of reinforcement type on the deformation at midspan. (**a**) deflection development; (**b**) curvature development.

Figure 6 displays the load-deflection and load-curvature graphs for axially restricted RC beams having different types of external reinforcement. These results are generated for the external reinforcement area of 1200 mm^2^ and depth of 500 mm. The results of the reference RC beam (i.e., beam without axial restriction) are also demonstrated in the graphs. The beams experience three different stages over the entire loading process. The turning points are caused by the cracking of concrete and yielding of bonded steel rebars. It is seen that axial restriction enhances the post-cracking stiffness, yielding and ultimate loads of RC beam but has no influence on the cracking load. The behavior of RC beam axially restricted with external CFRP reinforcement appears to be similar to that with external steel reinforcement. However, using external GFRP reinforcement is much less effective in improving the post-cracking stiffness and ultimate load than using external steel reinforcement. In this analysis, axial restriction by external steel, CFRP, and GFRP reinforcement increases the ultimate load by 77.3%, 65.3%, and 20.9%, respectively.

Flexural ductility is an important index representing the ability of inelastic deformation to dissipate seismic energy and to avoid premature failure. Flexural ductility may be quantified in terms of curvature ductility expressed by

(5) $$\mu_{\kappa }=\frac{\kappa_{u}}{\kappa_{y}}$$

where *μ_κ_* is the curvature ductility factor; *κ_u_* and *κ_y_* are curvatures at ultimate and first yielding, respectively.

It is seen that axial restriction leads to a reduction in flexural ductility of RC beam, especially when external steel or CFRP reinforcement is used. The beam axially restricted with external GFRP reinforcement shows better ductility than that with external steel or CFRP reinforcement. The curvature ductility factor of the reference RC beam is 5.83. After axial restricting by external steel, CFRP and GFRP reinforcement, the curvature ductility factor is reduced by 23.7% to 4.45, by 18.9% to 4.73, and by 4.6% to 5.56, respectively.

**Figure 7:**
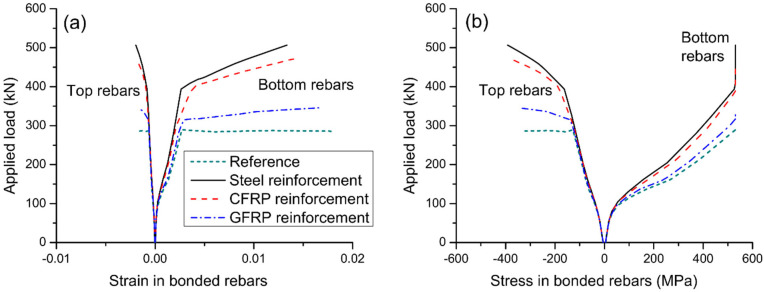
Effect of reinforcement type on the behavior of bonded rebars at midspan. (**a**) development of rebar strain; (**b**) development of rebar stress.

**Figure 8:**
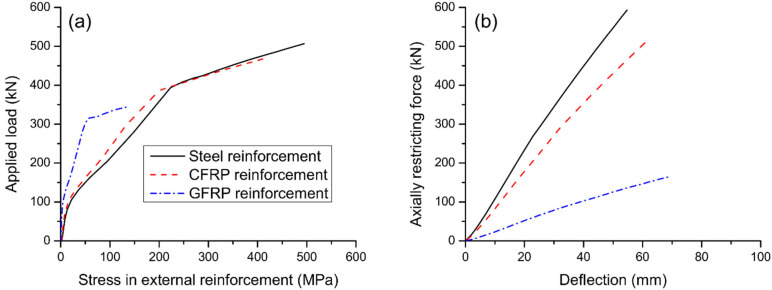
Effect of reinforcement type on the behavior of external reinforcement. (**a**) load versus reinforcement stress; (**b**) axially restricting force versus midspan deflection.

Figure 7 shows the influence of reinforcement type on strain and stress developments in internal steel rebars. It is noted that the bottom rebars (in tension) behave quite differently from the top rebars (in compression). The strain/stress development of bottom rebars is markedly affected by cracking and yielding while that of top rebars is affected by yielding only. At ultimate, the bottom rebars have reached their yield strength of 530 MPa while the top rebars have not yielded. Axial restriction reduces the tensile stress level in bottom rebars at a post-cracking service load while the stress reduction is less effective in utilizing external GFRP reinforcement compared to external steel/CFRP one. Figure 8a shows the stress development in different types of external reinforcement. Cracking and yielding lead to a quicker increase in stresses in external reinforcement. External CFRP reinforcement exhibits similar stress development to that of external steel reinforcement. At a given load level, external GFRP reinforcement shows markedly lower stress than the external CFRP or steel one. The ultimate stress in external steel reinforcement is 1.16 times that in external CFRP reinforcement, and 3.54 times that in external GFRP reinforcement. Figure 8b shows that there is an approximately linear relationship between axially restricting force and deflection. For the same deflection, external CFRP reinforcement causes a slightly smaller axially restricting force while external GFRP reinforcement results in a remarkably smaller axially restricting force, when compared to external steel reinforcement.

### 3.2. Effect of Reinforcement Area

**Figure 9:**
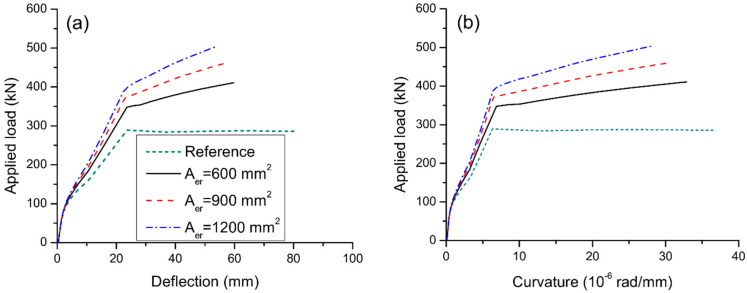
Effect of reinforcement area on the deformation at midspan. (**a**) deflection development; (**b**) curvature development.

Figure 9 displays the load–deflection and load–curvature graphs for axially restricted RC beams having different areas of external reinforcement. The results are produced using external steel reinforcement with a depth of 500 mm. As stresses in external reinforcement are negligible before cracking, the reinforcement area has practically no influence on the cracking load of the beams. After cracking, the contribution of external reinforcement is increasingly notable. Consequently, behavior of beams axially restricted with different areas of external reinforcement differs. A higher area of external reinforcement leads to stiffer behavior and higher yielding and ultimate loads. On the other hand, as the area of external reinforcement increases, the ultimate curvature and deflection decrease, causing a reduction of flexural ductility. In this analysis, axial restriction by external reinforcement with areas of 600, 900, and 1200 mm^2^ increases the ultimate load by 43.8%, 61.1%, and 77.3%, respectively, but reduces the curvature ductility factor by 13.0%, 19.0%, and 23.7%, respectively.

**Figure 10:**
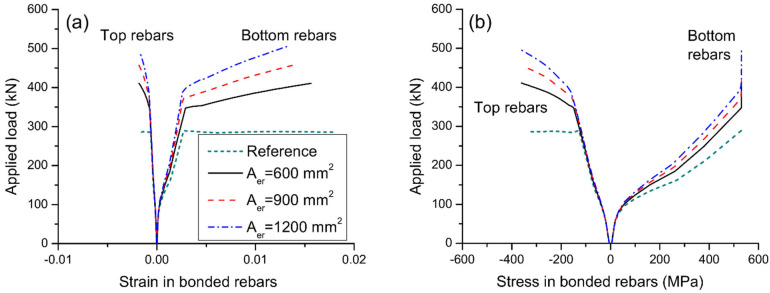
Effect of reinforcement area on the behavior of bonded rebars at midspan. (**a**) development of rebar strain; (**b**) development of rebar stress.

**Figure 11:**
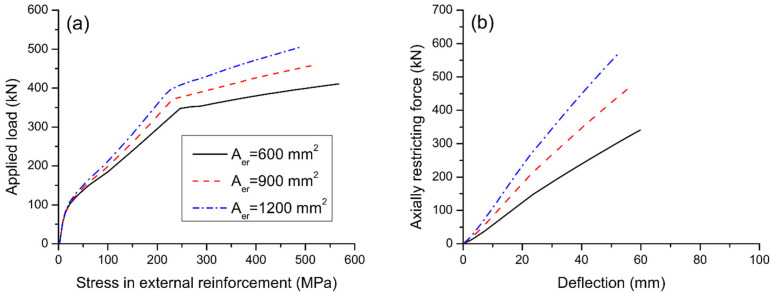
Effect of reinforcement area on the behavior of external reinforcement. (**a**) load versus reinforcement stress; (**b**) axially restricting force versus midspan deflection.

Figure 10 shows the effect of reinforcement area on strain and stress development of bonded steel rebars. At a given post-cracking load level, a higher area of external reinforcement results in a lower strain or stress in bottom rebars. As the area of external reinforcement increases, the ultimate strain in bottom rebars decreases while the ultimate strain or stress in top rebars tends to increase. Figure 11a shows the stress development of external reinforcement with different areas. At a given load level after cracking, the higher the area of external reinforcement, the lower the stress in external reinforcement. In addition, the ultimate stress in external reinforcement decreases as the reinforcement area increases. Figure 11b shows that the beam with a larger external reinforcement area mobilizes a higher axially restricting force at a given deflection or at the ultimate limit state, as expected. In this analysis, increasing A_er_ from 600 to 900 mm^2^ leads to a decrease in ultimate stress in external reinforcement by 12.9%, and an increase in axially restricting force at ultimate by 74.1%.

### 3.3. Effect of Reinforcement Depth

**Figure 12:**
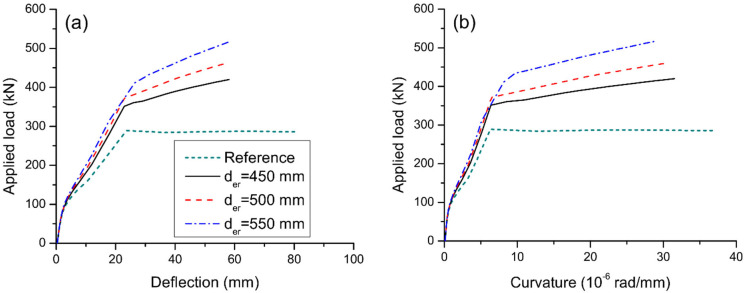
Effect of reinforcement depth on the deformation at midspan. (**a**) deflection development; (**b**) curvature development.

Figure 12 displays the load-deflection and load-curvature graphs for axially restricted RC beams having different depths of external reinforcement. The results are produced using external steel reinforcement with an area of 900 mm^2^. It is seen that a larger external reinforcement depth mobilizes greater yielding and ultimate loads. However, the flexural ductility tends to decrease as the depth of external reinforcement increases. In this analysis, axial restriction by external reinforcement with depths of 450, 500, and 550 mm results in an increase in ultimate load by 47.0%, 61.1%, and 81.3%, respectively, but a decrease in curvature ductility by 15.3%, 19.0%, and 22.7%, respectively.

**Figure 13:**
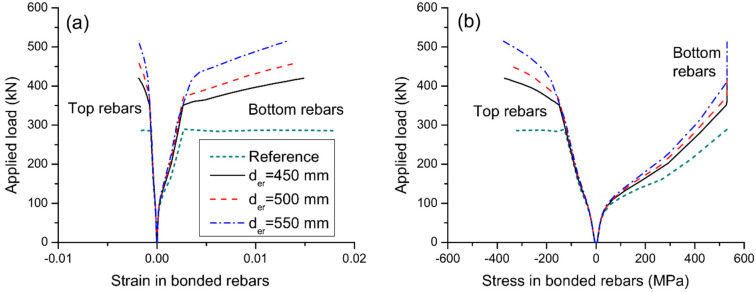
Effect of reinforcement depth on the behavior of bonded rebars at midspan. (**a**) development of rebar strain; (**b**) development of rebar stress.

**Figure 14:**
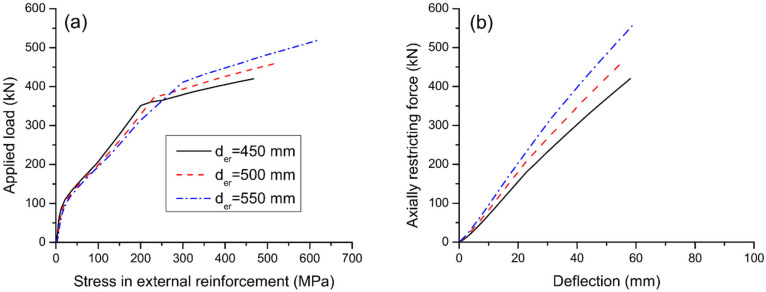
Effect of reinforcement depth on the behavior of external reinforcement. (**a**) load versus reinforcement stress; (**b**) axially restricting force versus midspan deflection.

Figure 13 shows the effect of reinforcement depth on strain and stress development of internal bonded rebars. It is observed that the reduction in strain or stress in bottom rebars appears to be more effective for a higher depth of external reinforcement. Also, a higher depth of external reinforcement causes smaller ultimate strains in bottom rebars but larger strains and stresses in top rebars. The effect of reinforcement depth on the load versus stress in external reinforcement is shown in Figure 14a. The change in strain in external reinforcement is dependent on the average strain change in the concrete at the same level of the external reinforcement along the entire beam span. A larger reinforcement depth corresponds to a larger average concrete strain at the same level of the external reinforcement, and thereby a larger stress in external reinforcement. The effect of reinforcement depth on the axially restricting force versus midspan deflection is shown in Figure 14b. At a given deflection level, a larger reinforcement depth leads to a higher axially restricting force. In this analysis, increasing d_er_ from 450 to 550 mm leads to an increase in axially restricting force (or stress in external reinforcement) at ultimate by 32.1%.

## 4. Theoretical Study on Ultimate Load Prediction

The prediction of the ultimate stress in external reinforcement (*σ_er_*) is a key step to determine the ultimate load of axially restricted RC beams. As external reinforcement is only connected to the concrete beam at the ends, there is no strain compatibility between external reinforcement and adjacent concrete. Therefore, the stress in external reinforcement is member-dependent, rather than section-dependent as in the case of internal bonded rebars.

**Figure 15:**
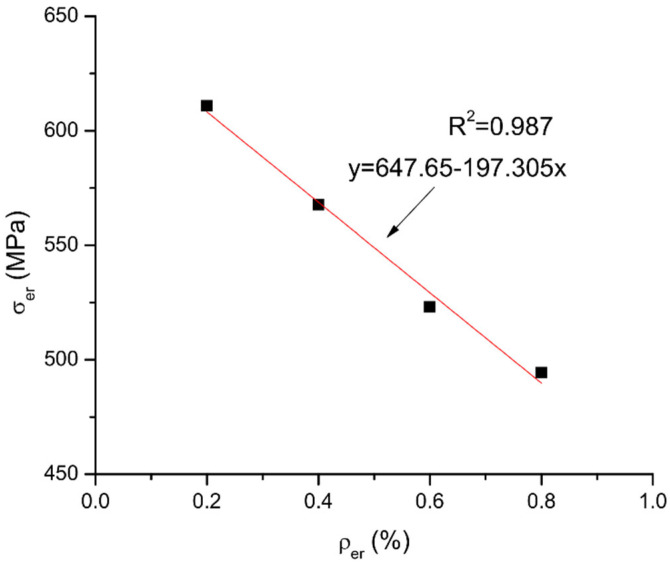
Linear fit to numerical data of *σ_er_* − *ρ_er_* relationship.

The parametric study presented in Section 3 shows that the ultimate stress in external reinforcement is influenced by three important parameters, i.e., the type, area and depth of external reinforcement. In this study, the external reinforcement ratio, *ρ_er_*, is adopted as a key parameter in a simplified equation to be developed for predicting the ultimate stress in external reinforcement. This parameter involves the area and depth of external reinforcement as expressed by

(6) $$\rho_{er}=A_{er}/(bd_{er})$$

where *b* is the section width. Figure 15 shows a linear fit to numerical data regarding the *σ_er_* − *ρ_er_* relationship of axially restricted RC beams. The numerical data are generated by using external steel reinforcement having various areas of 300–1200 mm^2^. The fit curve leads to the following equation:

(7) $$\sigma_{er}=648-19,700\rho_{er}$$

It is noted that the above equation is only valid for external steel reinforcement. As the ultimate stress in external reinforcement (*σ_er_*) is directly related to its elastic modulus (*E_er_*), Equation (7) can be extended to be also applicable to external FRP reinforcement by introducing the elastic modulus *E_er_*:

(8) $$\sigma_{er}=(648-19,700\rho_{er})E_{er}/200,000=(0.00324-0.0985\rho_{er})E_{er}$$

The axial equilibrium equation of the critical section of RC beams axially restricted with external reinforcement is

(9) $$0.85f_{c}b\beta_{1}c_{u}=A_{er}\sigma_{er}+A_{s}f_{y}-A_{s}^{\prime }f_{y}^{\prime }$$

where *β*_1_ is the stress-block factor for concrete, taken as 0.85; *c_u_* is the neutral axis depth at the ultimate limit state; *A_s_* and *f_y_* are the area and yield strength of tensile bonded steel rebars, respectively; $A_{s}^{\prime }$ and $f_{y}^{\prime }$ are the area and yield strength of compressive bonded steel rebars, respectively.

Substituting Equation (8) into Equation (9) results in

(10) $$c_{u}=\frac{A_{er}E_{er}(0.00324-0.0985\rho_{er})+A_{s}f_{y}-A_{s}^{\prime }f_{y}^{\prime }}{0.85f_{c}b\beta_{1}}$$

Hence, the ultimate moment (at midspan) of axially restricted RC beams evaluated at the level of external reinforcement is

(11) $$M_{u}=A_{er}E_{er}d_{eff}(0.00324-0.0985\rho_{er})+A_{s}f_{y}d_{s}-A_{s}^{\prime }f_{y}^{\prime }d_{s}^{\prime }-0.85f_{c}b{\left(\beta_{1}c_{u}\right)}^{2}/2$$

where *d_s_* and $d_{s}^{\prime }$ are depths of tensile and compressive bonded steel rebars, respectively; *d_eff_* is the effective depth of external reinforcement. The value of *d_eff_* can be calculated from

(12) $$d_{eff}=R_{d}d_{er}$$

where *R_d_* is the depth reduction factor as a result of second-order effects of axially restricted RC beams. According to Ref. [35], the value of *R_d_* may be calculated from

(13) $$R_{d}=0.87-0.01(L/d_{er})$$

where *L* is the span length.

For axially restricted RC beams under third-point loading, as illustrated in Figure 5, the ultimate load, *P_u_*, is then calculated from

(14) $$P_{u}=6(M_{u}-M_{d})/L$$

where *M_d_* is the moment induced by the dead load (self-weight).

**Figure 16:**
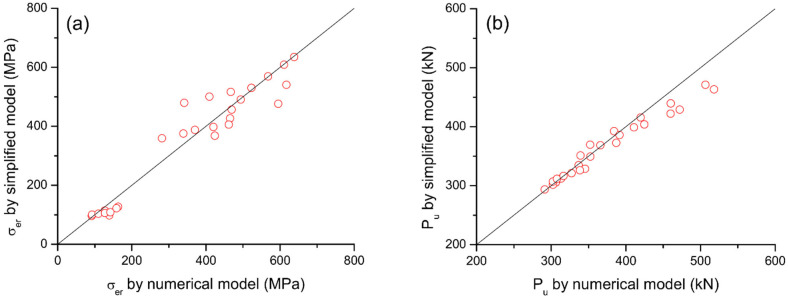
Correlation of results calculated by simplified and numerical models. (**a**) ultimate stress in externa reinforcement; (**b**) ultimate load.

A comparison of the ultimate stress in external reinforcement (*σ_er_*) and the ultimate load (*P_u_*) of RC beams axially restricted with different types, areas and depths of external reinforcement predicted by the proposed simplified model and numerical procedure is presented in Table 1 and Figure 16. Favorable agreement between the predictions by simplified model and numerical procedure is observed. The correlation coefficient between the *σ_er_* values obtained from simplified and numerical models is 0.964, and the mean discrepancy for *σ_er_* is −3.36% with a standard deviation of 15.39%. The correlation coefficient between the *P_u_* values obtained from simplified and numerical models is 0.984, and the mean discrepancy for *P_u_* is −2.11% with a standard deviation of 3.62%.

## 5. Conclusions

A numerical and theoretical study has been performed to evaluate the flexural behavior of RC beams axially restricted with external steel/FRP reinforcement. Based on the results of the present study, the following conclusions can be drawn:

- Axial restriction does not affect the pre-cracking behavior but has a marked impact on the post-cracking behavior of RC beams, i.e., axial restriction enhances the structural stiffness, yielding, and ultimate loads, but it leads to a reduction in flexural ductility.
- The type, area, and depth of external reinforcement influence substantially the ultimate stress in external reinforcement, and hence, the ultimate load of axially restricted RC beams. The ultimate stress in external reinforcement increases with the decrease of the reinforcement area or with the increase of reinforcement elastic modulus or depth.
- A simplified model is proposed to predict the ultimate stress in external reinforcement and the ultimate load of axially restricted RC beams, taking into account the effect of reinforcement type, area, and depth.
- The predictions by the simplified model agree favorably with the numerical results. For the ultimate stress in external reinforcement, the correlation coefficient is 0.964, and the mean discrepancy is −3.36% with a standard deviation of 15.39%. For the ultimate load, the correlation coefficient is 0.984, and the mean discrepancy is −2.11% with a standard deviation of 3.62%.

## Figures and Tables

**Table 1:** Comparison of ultimate stress in external reinforcement and ultimate load obtained from simplified and numerical models.

Reinforcement	*A_er_* (mm^2^)	*d_er_* (mm)	*σ_er_* (MPa)			*P_u_* (kN)		
			Numerical	Simplified	Error (%)	Numerical	Simplified	Error (%)
Steel	100	500	638.0	634.9	−0.48	308.9	310.6	0.55
	300		610.9	608.6	−0.38	352.5	349.3	−0.89
	600		567.7	569.2	0.27	410.9	398.9	−2.91
	900		523.1	529.8	1.28	460.3	439.2	−4.60
	1200		494.3	490.4	−0.78	506.7	471.0	−7.04
CFRP	100	500	595.3	476.2	−20.02	306.4	305.3	−0.37
	300		469.5	456.5	−2.77	336.8	334.6	−0.64
	600		465.2	426.9	−8.24	387.1	372.6	−3.74
	900		420.4	397.4	−5.49	424.8	403.8	−4.95
	1200		424.4	367.8	−13.34	472.3	428.8	−9.22
GFRP	100	500	163.1	127.0	−22.15	291.3	293.6	0.78
	300		159.0	121.7	−23.43	302.4	301.6	−0.25
	600		127.3	113.8	−10.59	313.4	312.2	−0.40
	900		127.6	106.0	−16.96	327.4	321.1	−1.92
	1200		139.5	98.1	−29.71	345.5	328.4	−4.96
Steel	900	350	341.8	479.1	40.19	352.2	369.5	4.92
		400	409.3	500.3	22.22	384.2	392.2	2.09
		450	467.1	516.7	10.61	420.0	415.5	−1.08
		500	523.1	529.8	1.28	460.3	439.2	−4.60
		550	617.2	540.5	−12.42	518.1	463.1	−10.61
CFRP	900	350	281.5	359.4	27.65	339.3	351.2	3.49
		400	339.2	375.2	10.61	365.8	368.4	0.69
		450	370.4	387.5	4.62	391.5	385.9	−1.41
		500	420.4	397.4	−5.49	424.8	403.8	−4.95
		550	461.7	405.4	−12.20	460.0	421.9	−8.28
GFRP	900	350	91.9	95.8	4.27	302.4	306.8	1.48
		400	92.9	100.1	7.68	307.7	311.5	1.24
		450	109.9	103.3	−6.01	316.6	316.3	−0.11
		500	127.6	106.0	−16.96	327.4	321.1	−1.92
		550	142.4	108.1	−24.09	338.6	326.0	−3.73

## Data Availability

Not applicable.
